# Occurrence and Biodegradation of Nonylphenol in the Environment

**DOI:** 10.3390/ijms13010491

**Published:** 2012-01-04

**Authors:** Zhen Mao, Xiao-Fei Zheng, Yan-Qiu Zhang, Xiu-Xiang Tao, Yan Li, Wei Wang

**Affiliations:** 1 School of Environment Science and Spatial Informatics, China University of Mining and Technology, Xuzhou 221116, China; E-Mails: xiaofeizheng11@163.com (X.-F.Z.); yqzhang@cumt.edu.cn (Y.-Q.Z.); lyhy_1969@163.com (Y.L.); wwwang0716@126.com (W.W.); 2 School of Chemical Engineering and Technology, China University of Mining and Technology, Xuzhou 221116, China; E-Mail: xiuxtao@163.com

**Keywords:** nonylphenol, endocrine disruptors, occurrence, biodegradation, water

## Abstract

Nonylphenol (NP) is an ultimate degradation product of nonylphenol polyethoxylates (NPE) that is primarily used in cleaning and industrial processes. Its widespread use has led to the wide existence of NP in various environmental matrices, such as water, sediment, air and soil. NP can be decreased by biodegradation through the action of microorganisms under aerobic or anaerobic conditions. Half-lives of biodegradation ranged from a few days to almost one hundred days. The degradation rate for NP was influenced by temperature, pH and additions of yeast extracts, surfactants, aluminum sulfate, acetate, pyruvate, lactate, manganese dioxide, ferric chloride, sodium chloride, hydrogen peroxide, heavy metals, and phthalic acid esters. Although NP is present at low concentrations in the environment, as an endocrine disruptor the risks of long-term exposure to low concentrations remain largely unknown. This paper reviews the occurrence of NP in the environment and its aerobic and anaerobic biodegradation in natural environments and sewage treatment plants, which is essential for assessing the potential risk associated with low level exposure to NP and other endocrine disruptors.

## 1. Introduction

Since the early 1990s, a lot of research has been performed concerning the endocrine disruptors which are widespread in the environment. Previous studies have demonstrated that nonylphenol (NP) is one of the endocrine disruptors, and many studies have shown that NP can exert adverse effects to an ecosystem.

NP is usually reacted to produce nonylphenol polyethoxylates (NPEs). NPEs are mainly used in a number of industrial processes and products, including cleaners, detergents and plastics. Annual world production of NPEs was about 520,000 tons in 1995, and the demand is increasing annually. In China, the production of NPEs is about 50,000 tons per year, and most enters into the aquatic environment [1]. The wide variety in use makes products containing NPEs potential sources of diffuse emissions of NPEs and NP. NPEs can biodegrade into NPs in sewage treatment works or in the environment. NP is persistent, lipophilic and tends to bioaccumulate more than the parent compounds [2,3]. Due to the endocrine potential of NP, the “Oslo and Paris Commission for the Protection of the Marine Environment of the north-east Atlantic” called for phasing out the use of NPEs in domestic cleaning agents by 1995 and in industrial cleaning agents by 2000. Following these recommendations, many countries, such as Sweden, Belgium, Great Britain, Germany, Holland, *etc*., have drastically limited the use of NPEs. Switzerland has completely banned the use of these substances [4]. In 2006, the U.S. Environmental Protection Agency (EPA) released final aquatic life ambient water quality criteria for NP, which recommends NP concentrations in both freshwater (28 μg/L, acute; 6.6 μg/L, chronic) and saltwater (7.0 μg/L, acute; 1.7 μg/L chronic). In Canada and Japan the use and production of NPEs are strictly monitored. But in many developing countries, such as China and India, no schedule was made to decrease the use of NP step by step. Meanwhile, the production of NPEs in these countries was increased annually.

## 2. Occurrence of Nonylphenol in the Environment

NP is a compound which has numerous isomers. The side chain has nine carbons and can be attached to phenol at different points on the ring, thus producing different isomers. 4-NP is the most common commercial forms of NP, which is often used in experimentation and the analysis [5,6]. At room temperature, NP is a pale yellow liquid with an approximate molecular weight of 215 to 220 g/mol and a specific gravity of 0.953 g/mL at 20 °C. It has a dissociation constant (pKa) of 10.7 ± 1.0 and an Octanol-Water Partition Coefficient (log Kow) between 3.8 and 4.8, and exhibits both pH- and temperature-dependent solubility, showing values of 6,350 μg/L at pH 5 and 25 °C [7].

NP is the biological breakdown products of widely used nonionic surfactant, NPEs, which is directly discharged into the environment. NP has been widely found in the environment: in water, sediment, air, soil, aquatic organisms and even human food.

### 2.1. Water and Sediment

#### 2.1.1. Surface Water and Sediment

Generally, NP occurs in the aquatic environment with concentrations varying widely in surface water from tens of ng/L [8,9] to dozens of mg/L [10]. In sediment, NP concentrations were higher than that of surface water. Table 1 shows the occurrence and distribution of NP in surface water and sediment in many countries. Because of the implementation of the European Directive 2003/53/EC, NP concentrations in European countries were much lower than in Asia.

Duong C.N. *et al.* [11] estimated the occurrence and distribution of NP in Korea and seven other Asian countries including Laos, Cambodia, Vietnam, China, Indonesia, Thailand and Malaysia. The results showed that the NP concentrations in samples of most Asian countries were at a higher level in comparison to those reported in European countries, America and Japan.

The domestic and industrial wastewater produced, as well as surface runoff, could possibly be sources of NP in the aquatic environment. The distribution and characteristics of pollution sources along the river affected the spatial variation of NP [1]. There was a direct relationship between concentrations of NP and the presence of urban or industrial activities near the sampling point [12]. Inadequately treated domestic wastewater caused high concentrations of NP in aquatic environment. Concentrations in the Kaoping River’s polluted tributaries were higher due to inadequate wastewater treatment in these regions [13], which caused high risk downstream of the river. The concentrations of NP in the Jialu River ranged from 75.2 to 1520 ng/L. Zhengzhou city is regarded as the main discharge source to this river as the annual discharge of NP from its urban zone to the river was 726 kg [14]. All of these results demonstrated that even a small river without adequately treated domestic wastewater could cause high mass loadings of NP and high risk [13]. In addition, many other factors affect the variation of NP in surface water, such as temperature, flow rate, and biodegradation *etc*. Temperature was the key factor affecting the seasonal variation of NP in water and suspended particles. In Seine River Estuary, NP maximum levels in the dissolved phase and in the suspended particulate matter were observed during winter periods while significant decreases were observed during spring and autumn periods [9]. These declines could be ascribed to maximum biological activities during these seasons. However, in terms of most water and suspended particles samples in Lanzhou Reach of Yellow River, concentrations were higher in warmer seasons than in colder seasons [1]. The higher NP concentrations in the warm season confirm the relationship between NP contamination and sewage discharge into the surface water, because more detergents, showers with shower cream, and plastic ware were used in the warm season compared to the cold one [15]. As to the flow rate, concentrations of NP in the water’s low flow period were higher than in its high flow period due to a low dilution factor [13,16].

Due to its hydrophobic properties, NP in surface water tends to be absorbed by sediment particles, which caused a preferential accumulation in sediments [8]. The reported sedimentary concentrations of NP were from a few μg/kg dry weight (dw) up to several hundred mg/kg dw (Table 1). The most contaminated sediment was found at Lake Donghu, China in 2003 [15]. NP was found at the highest concentration at 119,100 μg/kg dw. Similar circumstances were observed in Wuhan urban lakes [17] and Pearl River of China [18,19]. In Pearl River of China some researchers found there is a positive correlation between NP and total organic carbon (TOC), which indicated that sedimentary organic carbon (SOC) is a key factor in controlling the distributions of the endocrine disruptors [18,19]. Nowadays even in some European areas the NP concentrations in sediment were very high. For example, in the Danube River maximum sediment concentrations were 2,830 μg/kg dw for nonylphenol [20]. Table 1 shows the NP concentrations in samples of some countries in recent years. Generally speaking, the NP concentrations in sediment were at a higher level in developing countries than in developed countries.

High NP accumulation made the sediments a long-term pollutant sink and reservoir. The adsorbed compounds can be released into the water phase and become source of contaminants when hydraulic regimes of rivers change. However, De Weert *et al.* [21] thought that NP in the water phase was more available for biodegradation than in sediments. When the rate of biodegradation in the water is higher than the rate of desorption, NP is generally biodegraded in the sediment-water interphase and will not reach the bulk water, which may result in a limited hazard for the organisms in the aquatic environment. The effects of changing conditions on desorption and biodegradation processes are essential for adequate prediction of the fate of pollutants and the ecological effects of polluted sediments.

#### 2.1.2. Groundwater

Groundwater is of special interest because it makes up about twenty percent of the world’s fresh water supply and it is extraordinarily vulnerable to contamination by a variety of contaminants due to urban activities [36]. Micropollutants may enter the ground water nearly un-attenuated by bank filtration of affected surface waters or by infiltration or artificial recharge of treated wastewater into groundwater [37]. Contamination of groundwater is directly linked to the transport of the pollutant within the soil column supporting the advective and diffusional flow system, the geochemistry of the groundwater, and the overall groundwater flow [38].

Generally speaking, concentrations of NP in the groundwater were very low. In some area NP was not detected [38]. When NP was used as one of indicators for assessing anthropogenic impact on urban surface and groundwater in the cities of Halle/Saale and Leipzig (Germany), concentrations of NP were observed about 100 ng/L [39]. Loos R. *et al.* [37] collected and analyzed 164 individual groundwater samples from 23 European Countries. NP was found in 11% of the samples (with a LOD of 30 ng/L), with a maximum concentration of 3.8 μg/L, exceeding the European groundwater quality standard for pesticides of 0.1 μg/L for several samples. In Austria, the most abundant industrial chemicals in groundwater samples were NP, occurring in about half of the samples. The maximum concentration of NP was 1,500 ng/L, and the 90th percentile of NP was 424 ng/L [40]. The NP pollution of groundwater may threaten human and ecosystem health.

#### 2.1.3. Drinking Water

Recently, drinking water safety has received significant attention. Contaminants, such as NP, in drinking water might pose health risks to some residents. NP were detected in bottled water with the concentration of about 7.9 ng/L [41]. Li X. *et al.* [42] investigated the 4-NP level in tap water in Guangzhou (China) using gas chromatography–mass spectrometry with negative chemical ionization. Five of the tap water samples from six drinking water plants were found to contain 4-NP both in June and December. The highest concentration in tap water for 4-NP was 1,987 ng/L. In Chongqing, another major city of southwestern China, the 4-NP removal rate by the water treatment process varied in a range from 62% to 94%, resulting in a considerably high residual 4-NP concentration in drinking water in July (0.1–2.7 μg/L) [43]. Although the daily intake values of 4-NP for a human is much lower than their tolerable daily intake (TDI) values, which are 5 μg/kg body weight for NP [42], more attention should be paid to the ecological risk.

#### 2.1.4. Wastewater Treatment Plants

NP are the most abundant compounds in raw wastewater as well as in effluents from all the treatment stages of sewage treatment plants. In influent wastewater, concentrations of NP ranging from 0.08 to 96.4 μg/L [44–49] have been reported by investigators. Biodegradation was the main removal pathway of NP, as it was more effective in removing NP from the aqueous phase than physical treatment [50]. A wide range of microorganisms were involved in NP biodegradation via different degradation pathways, which reduced the possible risk of NP in the environment under aerobic conditions [51]. Removal rates of NP ranging from 13.6 to >99% have been reported in literature [50,52–54]. Generally, the elimination efficiency varied between 73% and 92% [46]. In the European Union Wastewater Treatment Plants (WWTPs) 4-NP has shown remarkable decreasing influent and effluent concentrations since the implementation of Directive 2003/53/EC [46].

In a wastewater treatment plant many factors can influence the removal of NP, such as influent load, water quality of influents, plant configurations, hydraulic residence time (HRT), sludge retention time (SRT), biomass characteristics and the environmental conditions [50,52,53,55,56]. Longer HRT or SRT and greater microbial activity appear to have a positive influence on the ability of the activated sludge system to eliminate NP [50,57]. At aerobic conditions the NP degradation potential was affected by changes in pH value or temperature, and by the addition of yeast extract, aluminum sulfate, hydrogen peroxide or surfactant [58]. Nie *et al.* thought that warm temperature and a high MLSS concentration would benefit the removal of NP from wastewater in the Anaerobic/Anoxic/Oxic (A/A/O) bioreactor [59].

During wastewater treatment, NP accumulated in sewage sludge at a concentration of several hundred mg/kg [48,60,61]. Because NP may be highly toxic to organisms, further research is needed to evaluate their degradability and set up disposal procedures to minimize the environmental impact produced by the use of sludge for agricultural purposes [48].

### 2.2. Air

The occurrence of NP in the air was ubiquitous in urban, remote, industrialized, coastal regions, and even in the central part of the North Sea (Table 2). NP is not produced naturally so their presence in the environment is the consequence of anthropogenic activity [62]. As NP present short atmospheric life they are not considered as environmentally persistent or subjected to significant long-range transport. Wind direction did not show significant influence on NP concentration. Therefore the atmospheric NP originated from local sources [63]. For this reason, the concentration of NP was higher in a densely populated and more polluted urban area [64] than remote area [65]. As a semivolatile organic compound, NP can vaporize into atmosphere from wastewater discharges, wastewater treatment plant effluents (liquid and sludge) or polluted surface waters [62,63]. The NP occurrence in the atmosphere may be an important human and ecosystem health issue in the world [64].

The atmospheric concentration of NP showed declining trends from land to the open sea, suggesting that the atmosphere is a significant pathway for the transport of alkylphenols in the environment [67]. Sea could be an important sink for the NP, and might be as a potential source for the occurrences of NP in the oceans and remote area [68]. In the winter, atmospheric deposition was dominant. However in the warm seasons, re-volatilization might happen [68]. The similar seasonal trends were observed in the lower Hudson River Estuary [66]. At all the sampling sites, gas-phase NP concentrations were significantly higher during the summer (June–September) than during the fall and early winter (October–December). Temperature might be a critical factor contributing to the seasonal trends of NP in atmosphere.

### 2.3. Soil

NP can be introduced into soils in various ways, for example from atmospheric deposition, from soil amendment with sewage sludge, and from wastewater for irrigation of agricultural land [69]. During wastewater treatment, large quantities of NP can be quickly sorbed by the organic rich solid phase and eventually concentrated in biosolids with levels from a few mg/kg up to several thousand mg/kg [70,71]. Biosolids are often used as fertilizer on agricultural soils and additionally it improves soil structure and aids the recycling of nutrients and organic matter [72]. NP could be introduced to soils via land application of biosolids, potentially leading to accumulation in soils and crops. NP was detected at μg/kg levels in the biosolids-amended soils [73] and the wastewater irrigated soils [74].

Although NP is capable of being leached from soil, its short half-life means that its passage from soil to freshwater will be low [75]. Previous studies showed that no NP will accumulate over time and plant uptake or water quality impairment will be minimal [75–77]. This suggests that NP in the soils most probably pose very low risks to the soil and freshwater ecosystems and even human health [74,75].

## 3. Biodegradation of Nonylphenol in the Environment

NP can be decreased by biodegradation in the water, sediment and soils through the action of microorganisms. In the Jialu River, about 23.7% of total decrease in NP concentration was caused by biodegradation [14]. As there are many chemical and environmental factors which influence biodegradation of NP, half-lives (t1/2) for NP aerobic degradation in sewage sludge and sediments ranged from 1.1 to 99.0 days [78–80]. Aerobic degradation rate for NP was enhanced by shaking, increased temperature and the addition of yeast extract (5 mg/L) and surfactants such as brij 30 or brij 35 [78–80]. The addition of aluminum sulfate, hydrogen peroxide, Pb, Cd, Cu, Zn, phthalic acid esters (PAEs), and NaCl inhibited NP degradation [78,80]. Reduced levels of ammonium, phosphate, and sulfate also delayed the aerobic degradation rate for NP [79]. And the optimal pH value for NP biodegradation was 7.0 [78]. Biodegradation ability was also related to light intensity in some microorganisms, such as *C. vulgaris* [81].

NP degradation under anaerobic conditions has only recently been demonstrated. Half-lives of anaerobic degradation ranged from 23.9 to 69.3 days [82,83]. Anaerobic degradation rate for NP was enhanced by increasing temperature and the addition of yeast extract or surfactants such as brij 30 or brij 35. The addition of aluminum sulfate, acetate, pyruvate, lactate, manganese dioxide, ferric chloride, sodium chloride, heavy metals, and phthalic acid esters inhibit the degradation rate. The high-to-low order of degradation rates was: sulfate-reducing conditions > methanogenic conditions > nitrate-reducing conditions [82,83].

Upon entering soil, NP can undergo a number of reactions (e.g., sorption, biodegradation, leaching, plant uptake) that ultimately control its fate and potential environmental hazard [75]. The concentrations of NP sharply declined with increasing soil depth, indicating limited soil leaching of this compound [73]. Previous studies showed that different NP isomers exhibited different degradation rates, but only minimal amounts of all isomers persisted after 45 d [76,84]. Das and Xia found that isomers with a-methyl-a-propyl structure transformed significantly slower than those with less branched tertiary a-carbon and those with secondary a-carbon [70]. The main factors controlling the degradation and the rate of degradation are initial concentration of NP, soil parameters, environmental conditions, as well as agricultural practices [84,85]. Trocme *et al.* showed that NP degraded rapidly during incubation at low concentrations, but was more persistent at higher concentrations [86]. Appropriate pH value, temperature and the aeration status in soils contribute to increase microbial activity, and consequently enhance NP degradation. The addition of different substrates such as yeast extract, compost, or brij 35 also changed the microbial community and thus affected NP degradation in soil [87]. Some treatments, such as sludge centrifugation and lime stabilization, decrease the rate of mineralization and significantly lower degradation [85].

During recent years, a lot of research has been performed concerning the molecular mechanism for degradation of nonylphenol by a number of different strains. Understanding in more detail the molecular events in degradation of nonylphenol were illuminated from two different pathways. The ring cleavage pathway was elucidated through gene cloning and biochemical studies [88,89], which involves two critical steps. First, the aromatic ring was monohydroxylated by a multicomponent phenol hydroxylase at the ortho position. Then aromatic ring was cleaved either by catechol 1,2-dioxygenase (C12O) (which is responsible for the ortho-pathway) or catechol 2,3-dioxygenase (C23O) (which is responsible for the meta-pathway). Zhang *et al.* [88] investigated the changes of possible key catabolic genes during the degradation of NP in natural water microcosms and found that the copy number of catechol 2,3-dioxygenase (C23O) DNA increased significantly during NP degradation. This result suggested that meta-cleaving pathway might be involved in the degradation of NP natural water microcosms. However, Nguyen *et al.* [89] found that most of the isolated alkylphenol-degrading bacteria are able to degrade long-chain alkylphenols via multicomponent phenol hydroxylase and the ortho-cleavage pathway. The ipso-hydroxylation pathway was responsible for the removal of the alkyl chain from NP by Sphingomonas strains [90–96], in which NP isomers were initially hydroxylated at the ipso-position forming dienones, and subsequently the nonyl chain shifts to the oxygen atom in the introduced hydroxyl group to form alkoxyphenols, from which the alkyl moieties can be easily detached as alcohols by known mechanisms [95,97].

## 4. Conclusions

NP is a virtually ubiquitous contaminant in the environment. The occurrence of NP has been reported around the world in waters, sediment, airs and soils. It can be decreased by biodegradation in natural environments and sewage treatment plants through the action of microorganisms under aerobic or anaerobic conditions. Although NP is present at low concentrations, the risks of long-term exposure to low concentrations remain largely unknown. More research needs to be done to determine the potential human and environmental health risks posed by exposure to NP in the environment.

## Figures and Tables

**Table 1:** Nonylphenol (NP) levels in surface water and sediment samples.

NP levels				
			
Surface water (μg/L)	Sediment (μg/kg)	Location	Detected time	Reference
0.034–0.599	38.4–863.0	Lanzhou Reach of Yellow River, China	July and November 2004	[1]
NA-0.53	LOD-79	Llobregat basin, Spain	2005–2006	[8]
0.112	266	Thermaiko Gulf, Greece		[22]
0.227	-	Loudias River, Greece		[22]
<LOD-310	-	Kaoping River and its tributaries, Taiwan	July 2004–December 2005	[13]
0.075–1.520	-	Jialu River, China September	2007	[14]
0.266 ± 0.028	-	Yeongsan and Seomjin rivers, Southern Korea	2008	[11]
0.043 ± 0.005	-	Ton River in Souan Mone, Pear Lart and Park Ton, Laos	2008	[11]
< LOD	-	Siem Reap River, Chong Srok area, Cambodia	2008	[11]
2.097 ± 0.212	-	Long Xuyen city and nearby area, Vietnam	2008	[11]
0.372 ± 0.040	-	Fenhe River, China	2008	[11]
0.039 ± 0.005	-	Cikamasan, Cisarua, Indonesia	2008	[11]
0.918 ± 0.103	-	Khong River, Thailand	2008	[11]
0.814 ± 0.089	-	Tuaran, Salut River area, Malaysia	2008	[11]
<0.029–0.195	-	Glatt River, Switzerland	September 2006	[23]
1.94–32.85	3,540–32,430	Wuhan urban lakes, China	October 2005	[17]
<0.1–1.4	44–567	Rieti district, Italy	2002, 2003	[12]
0–0.24	-	Danube River	August–September 2007	[24]
0–1.40	-	Tributaries of Danube River	August–September 2007	[24]
-	<20–2,830	Danube River	2007	[20]
<0.029–0.233	-	Ria de Aveiro, Portugal	August 2006	[25]
LOD-0.015	LOD-1,750	Great Lakes coastal wetland, Cootes Paradise, Canada	2001, 2002	[26]
75.2–179.6	5,460–119,100	Lake Donghu, China	April 2003	[15]
0.036–33.231	-	Pearl River Delta, China	2005, 2006	[10]
0.152–13.757	-	Thessaloniki, Greece	2005–2006	[27]
0.015–0.386	-	Seine River Estuary, France	2002–2004	[9]
<LOD-0.770	-	Hessisches Ried region, Germany	2003–2006	[28]
<LOD-0.511	-	Donggang River, Taiwan	2002	[16]
0.1–0.5	75–340	Cuyahoga River, Ohio, USA		[29]
<0.210	<350	Minnesota lakes, USA	2008	[30]
0.068–0.326	-	Glatt River, Switzerland	2004	[31]
-	107–16,198	Pearl River system, China	2006–2007	[18]
-	13–225	Bay of Cadiz, Spain	2002	[32]
-	31–21,885	Pearl River Delta, China	2006–2007	[19]
	3.1	Dianchi Lake, China		[33]
	<LOD-1,364	Upper Danube River	2006	[34]
	47–192	Venice lagoon, Italy	2001–2002	[35]

LOD: Detection limit. NA: not analyzed.

**Table 2:** NP levels in air.

NP levels (ng/m^3^)		Location	Detected time	Reference

Gas	Aerosol			
19.2 (1.5–69)	6.1 (0.1–14)	Hudson River Estuary, USA		
10.2 (0.9–56)	9.8 (0.3–51)	Sandy Hook, USA	June–October, 1998	[64]
2.5 (0.2–8.1)	5.6 (1.8–23)	Liberty Science Center, USA		
6.9 (nd-56)	5.4 (0.067–51)	Sandy Hook, USA		
2.6 (nd-17)	3.8 (0.23–23)	Liberty Science Center, USA	June–December 1998	[66]
13 (0.13–81)	0.55 (0.020–6.4)	New Brunswick, USA		
1.60–16.5	-	Urban site of Thessaloniki, Greece	January–February 2007	[63]
0.15–1.0	0.0017–0.117	NE-Bavaria, Germany	May–November 2001	[65]
0.22 (0.055–0.42)	0.040 (0.010–0.12)	GKSS Research Centre, Germany	-	
0.056 (0.029–0.11)	0.010 (0.005–0.017)	North Sea	-	[67]
About 0.01–0.1	-	North Sea	February–March 2004	[68]
